# Genetic Susceptibility in Dry Eye Disease: Immunogenetic Evidence, Current Limitations, and Future Directions

**DOI:** 10.3390/genes17101253

**Published:** 2026-10-10

**Authors:** Matteo Capobianco, Marco Zeppieri, Francesco Cappellani, Caterina Gagliano, Roberto Angelo Consoli, Sonia Mangiagli, Anna M. Roszkowska

**Affiliations:** 1 Eye Clinic, Catania University, Policlinico G. Rodolico, 95121 Catania, Italy; capobiancoteo@gmail.com; 2 Department of Ophthalmology, University Hospital of Udine, 33100 Udine, Italy; 3 Department of Medicine, Surgery and Health Sciences, University of Trieste, 34129 Trieste, Italy; 4 Department of Medicine and Surgery, University of Enna “Kore”, Piazza dell’Università, 94100 Enna, Italy; 5 Mediterranean Foundation “G.B. Morgagni”, 95125 Catania, Italy; 6 Department of Biomedical Sciences, University of Messina, 98122 Messina, Italy; 7 Faculty of Medicine, Andrzej Frycz Modrzewski Krakòw University, Gustawa Herlinga-Grudzińskiego 1, 30-705 Krakow, Poland

**Keywords:** dry eye disease, ocular surface, ophthalmic genetic susceptibility, genome-wide association studies, heritability, polygenic risk scores, HLA, single-cell transcriptomics, precision medicine

## Abstract

Dry eye disease is a multifactorial disorder which is heterogeneous in nature, caused by interactions among ocular surface abnormalities, systemic diseases, environmental factors and host susceptibility. The inherited variation related to the pathophysiology of dry eye disease is not very well characterized, but known mechanisms include inflammation, tear film instability, neurosensory dysfunction and lacrimal and meibomian gland changes. Studies exploring the contribution of genetics suggest that dry eye disease (DED) and specific ocular surface endophenotypes are heritable. Candidate-gene and human leukocyte antigen association studies have reinforced contributions from pathways associated with innate and adaptive immunity, inflammatory regulation and neural function, and glandular homeostasis, but many associations require replication in larger, more diverse populations. Genome-wide association studies and biobank-based assessments have recently begun to shed light on the polygenic architecture of Dry Eye Disease (DED) and its genetic overlap with other related conditions worldwide. This paper evaluates critically the current evidence on DED heritability, candidate susceptibility genes, HLA associations, genome-wide signals, polygenic risk models and genetic pleiotropy. It will cover how transcriptomic, epigenomic, and single-cell approaches can link inherited susceptibility loci to disease-relevant cell states and molecular pathways across the ocular surface unit, including the cornea, conjunctiva, lacrimal gland, and meibomian glands. The study would stress phenotypic heterogeneity, ancestry-related limitations, and inconsistent diagnostic definitions. In the end, we will evaluate opportunities and existing constraints in the incorporation of genomic information with clinical endophenotypes and environmental exposures to support risk stratification, biomarker discovery, identification of therapeutic targets, and the development of future ocular surface precision medicine.

## 1. Introduction

Dry eye disease (DED) is a multifactorial and heterogeneous disorder of the ocular surface in which symptoms arise in association with loss of tear film and/or ocular surface homeostasis. Contemporary definitions recognize tear film instability and hyperosmolarity, ocular surface inflammation and damage, and neurosensory abnormalities as major etiological contributors rather than mutually exclusive mechanisms [1,2]. The most recent Tear Film & Ocular Surface Society Dry Eye Workshop (TFOS DEWS III) further emphasizes that DED encompasses biologically diverse disease states whose clinical expression may vary according to age, sex, systemic disease, ocular comorbidities, environmental exposures, and individual susceptibility [3]. At the population level, DED is associated with numerous demographic, systemic, pharmacological, and lifestyle-related factors, illustrating the substantial contribution of the exposome to disease risk [4,5].

Despite major advances in understanding inflammation, epithelial dysfunction, lacrimal and meibomian gland abnormalities, tear film instability, and altered corneal somatosensory signaling, considerably less is known about the inherited component of DED. Earlier reviews highlighted the possibility that variation in genes regulating mucosal barrier function, immunity, inflammation, neural signaling, and glandular physiology could influence susceptibility to DED, while also emphasizing the scarcity of adequately powered genetic studies [6]. More broadly, genetic disorders affecting the ocular surface demonstrate that epithelial integrity, innervation, immune regulation, and tear-producing tissues are highly dependent on genetically regulated pathways [7]. However, the genetic architecture of common multifactorial DED is expected to differ substantially from that of monogenic ocular surface disorders and is likely to involve numerous variants of small effect interacting with age, sex, systemic disease, and environmental exposure.

Evidence for a heritable component of DED remains limited. A large female twin cohort reported heritability estimates for DED diagnosis and several ocular-surface traits, but these estimates were phenotype-dependent and have not been broadly replicated [8].

This heterogeneity is particularly relevant because DED is not a single biological phenotype.

Symptoms, aqueous tear production, tear-film stability, meibomian gland dysfunction, inflammatory measures, neuropathic-like ocular pain, and severity indicators such as ocular-surface staining may vary independently or occur in partially overlapping combinations.

Consequently, a binary diagnosis of “DED” may combine genetically distinct endophenotypes and reduce power in genomic studies.

The emergence of large biobanks, genome-wide association studies (GWASs), polygenic risk scores (PRSs), transcriptomics, epigenomics, and single-cell technologies has created an opportunity to revisit the genetic basis of DED at substantially greater resolution. Nevertheless, the evidence remains uneven. Candidate-gene studies have generally included relatively small and ancestry-restricted cohorts, direct GWAS of DED remain limited compared with those of age-related macular degeneration, glaucoma, or myopia, and some of the largest currently available genomic analyses have not yet undergone peer review.

Systemic autoimmune diseases associated with ocular dryness, particularly Sjögren disease, have a more extensively studied but still incomplete and polygenic immunogenetic background; these findings cannot be assumed to represent the genetic architecture of non-Sjögren DED.

This review evaluates four complementary levels of evidence: (1) heritability and clinically defined DED phenotypes; (2) candidate-gene and HLA/immunogenetic associations; (3) genome-wide associations, polygenic risk, pleiotropy, and gene–environment relationships; and (4) functional molecular studies used to interpret putative susceptibility pathways. Throughout, evidence is assessed according to phenotype definition, sample size, ancestry, replication, publication status, and directness of relevance to DED. We also distinguish germline genetic susceptibility from disease-associated transcriptional, epigenetic, and non-coding RNA changes.

## 2. Materials and Methods

The objective of this work was to present a structured narrative review of the DED genetic architecture. The PubMed/MEDLINE search was performed on 27 August 2026 with no predefined publication-date restriction. In addition to this, publisher records were consulted to confirm bibliographic information, online-ahead-of-print publication, and publication status of relevant records. The search combined terms referring to DED and the ocular surface with terms referring to genetic susceptibility and functional genomics. We focused our search on a number of keywords when looking at the literature relating to the genetic susceptibility of dry eye disease. The principal search structure was: (“dry eye disease” OR “dry eye syndrome” OR “keratoconjunctivitis sicca” OR “ocular surface”) AND (“genetics” OR “genetic susceptibility” OR “heritability” OR “twin” OR “single nucleotide polymorphism” OR “SNP” OR “candidate gene” OR “HLA” OR “genome-wide association study” OR “GWAS” OR “polygenic risk score” OR “genetic correlation” OR “pleiotropy” OR “Mendelian randomization” OR “transcriptomics” OR “epigenetics” OR “DNA methylation” OR “microRNA” OR “non-coding RNA” OR “single-cell RNA sequencing”). Essentially, we searched for articles looking at the genetic aspect of DED and how it can affect the ocular surface. Sjögren disease was additionally considered for studies that provided genetic or molecular information directly related to lacrimal gland dysfunction, keratoconjunctivitis sicca, or autoimmune ocular surface disease.

Studies that specifically focused on inherited susceptibility to DED, heritable ocular-surface phenotypes, genetic association, genome-wide architecture, polygenic risk, genetic correlation, and/or Mendelian randomization or functional molecular mechanisms relevant to interpretation of DED-associated genetic pathways were screened. We first looked at the titles and abstracts for relevance, followed by the full text of potentially eligible reports. Human genetic association studies, twin studies, GWAS and biobank analyses, well-characterized cohort studies, and recent peer-reviewed reviews or consensus reports from major ophthalmic and biomedical journals were prioritized. Older primary studies were retained when they provided foundational evidence for specific candidate-gene associations or heritability estimates that were lacking modern equivalents. Experimental animal studies were included selectively when they provided functional genomic, epigenomic, or single-cell evidence relevant to the interpretation of human DED biology. Studies relating to Sjögren disease were only included when directly relevant to ocular dryness or lacrimal-gland biology and interpreted as evidence for an autoimmune DED endotype, not as evidence applicable to DED as a whole. Records without direct relevance to DED or ocular-surface biology, duplicate reports, and retracted publications were omitted.

Given the large heterogeneity in DED definitions, genetic exposures, molecular assays, ancestry, and reported outcomes, no quantitative meta-analysis was performed. Evidence was synthesized by study design, sample size, directness of relevance to the DED phenotype or endotype, strength of statistical association, replication status, and publication status. Particular attention was devoted to differentiating between associations that achieve genome-wide significance and those that are suggestive. We distinguish candidate-gene findings from replicated genomic associations, germline variation from disease-associated transcriptional or epigenetic changes, and evidence from peer-reviewed literature from preliminary reports or preprints. Molecular and functional studies were used to support biological interpretation and were not considered evidence of inherited susceptibility unless there was direct evaluation of germline variation. As this review is not a systematic review, it should not be considered a comprehensive list of all genetic studies evaluating DED.

As this article synthesizes previously published data and does not involve new human or animal experimentation, institutional ethical approval and informed consent were not required.

## 3. Results

### 3.1. Evidence for Genetic Predisposition in DED: Limited and Phenotype-Dependent

Twin studies suggest a possible inherited contribution to selected DED phenotypes, but the evidence remains limited and phenotype-dependent.

In 3930 female monozygotic and dizygotic twins from the TwinsUK registry, Vehof et al. estimated a heritability of 29% for dry-eye symptoms and 41% for a clinician diagnosis of DED accompanied by artificial-tear use. Greater variation was observed among objective and semi-objective ocular-surface traits assessed in a subset of 606 twins. Heritability estimates were 25% for interblink interval, 40% for tear osmolarity, 58% for Schirmer test values, and 78% for the presence of blepharitis, whereas no significant genetic contribution was detected for tear breakup time [8].

These estimates suggest that familial or genetic contributions may vary across ocular-surface traits, but they derive from a single female twin cohort and should not be generalized to all DED populations or endotypes.

Heritable variation may also be present at the molecular level of the tear film. Bjerager et al. examined 90 healthy twins comprising 23 monozygotic and 22 dizygotic pairs and estimated broad-sense heritability for 15 tear-fluid cytokines. Ten showed heritability estimates above 50%, with particularly high estimates for interferon-γ-induced protein 10 (94.8%), eotaxin (89.8%), IL-7 (86.6%), IL-1β (82.2%), and monocyte chemoattractant protein 1 (68.2%) [9].

These observations suggest that baseline concentrations of some tear inflammatory mediators may partly reflect inherited variation in healthy individuals.

However, the study was conducted in healthy individuals and does not demonstrate that genetically determined cytokine concentrations increase the risk of DED.

These findings therefore describe interindividual variation in healthy twins and should not be interpreted as evidence of genetic susceptibility to DED.

The symptom component of DED may incorporate another genetically influenced dimension related to pain perception. In a population-based study of female twins, Vehof et al. assessed generalized pain sensitivity using heat pain threshold and suprathreshold testing. Women classified as having DED exhibited lower heat pain thresholds and lower pain tolerance than those without DED, and pain-related Ocular Surface Disease Index symptoms were significantly associated with both measures. Participants with a heat pain threshold below the population median reported DED pain symptoms more frequently than those above the median (31.2% versus 20.5%; OR 1.76, 95% CI 1.15–2.71) [10].

This study assessed pain sensitivity rather than genetic variation or heritability of ocular pain and therefore provides only indirect evidence relevant to genetic susceptibility in DED.

A larger twin analysis explored possible shared familial and genetic influences between dry eye and several chronic pain phenotypes.

Among 8564 twins evaluated for dry eye, chronic widespread musculoskeletal pain, chronic pelvic pain, migraine, and irritable bowel syndrome, monozygotic correlations were consistently greater than dizygotic correlations, supporting heritable contributions to each phenotype. With migraine excluded from the common-pathway analysis, cross-trait modeling supported shared genetic influences among dry eye, chronic widespread pain, pelvic pain, and irritable bowel syndrome; the latent factor underlying these correlated conditions had an estimated heritability of 66% [11]. This finding does not establish that DED is primarily a pain disorder or identify a specific genetic mechanism for neuropathic ocular pain.

The analysis therefore suggests possible shared genetic influences across these phenotypes, but it does not establish a DED-specific genetic mechanism and should be considered indirect evidence.

Collectively, these studies indicate that genetic susceptibility to DED cannot be adequately represented by a single heritability estimate.

Clinical diagnosis, symptoms, tear-production measures, tear osmolarity, inflammatory mediators, and pain-related traits have yielded heterogeneous estimates of possible inherited contribution across different populations and phenotype definitions [8,9,10,11].

This phenotype dependence supports the use of quantitative endophenotypes alongside conventional case–control definitions in genetic studies of DED, particularly when attempting to separate glandular, tear-film, inflammatory, and sensory contributions to a clinically heterogeneous disorder.

### 3.2. Candidate Susceptibility Genes and HLA-Associated Immune Architecture

Throughout this section, associations are attributed to the specific phenotype or clinical context in which they were identified; findings restricted to Sjögren-associated DED, post-refractive-surgery ocular surface disease, or other specific endotypes are not interpreted as general genetic risk factors for DED.

Early candidate-gene studies selected loci because of their putative biological relevance to epithelial, inflammatory, or immune pathways; however, biological plausibility of a pathway does not itself establish genetic susceptibility to DED.

MUC1 provided one of the first examples linking ocular-surface biology with potential inherited susceptibility. Analysis of human corneal, conjunctival, and lacrimal tissues identified multiple MUC1 splice variants, while conjunctival brush cytology suggested that the proportion of dry-eye patients expressing the MUC1/A variant was lower than in normal controls. MUC1/A was associated with alleles containing larger variable numbers of tandem repeats (VNTRs), leading to the hypothesis that differences in MUC1 structure might influence ocular-surface protection [12]. Subsequent genotyping refined this observation: the rs4072037 polymorphism controlling MUC1/A versus MUC1/B splicing was perfectly correlated with splice-variant expression in the samples examined, and genotype frequencies differed between female Caucasian controls and both non-Sjögren aqueous-deficient DED with ocular-surface staining and evaporative DED. In contrast, the proposed relationship with VNTR size was weaker than anticipated, and a previously reported estrogen receptor-α association was not reproduced in this population [13].

The MUC1 findings remain hypothesis-generating because they derive from small candidate-gene studies and have not been confirmed in large independent genomic datasets [12,13].

Candidate analyses of inflammatory genes have yielded similarly specific but incompletely replicated signals. In Korean patients with non-Sjögren DED, the IL1B rs1143634 polymorphism differed between cases and controls, with the C/T genotype reported in 10.4% of patients compared with 3.9% of controls (OR 3.337). The IL6R Asp358Ala polymorphism rs8192284 was also associated with DED, including the C/C genotype (OR 2.12), whereas no significant association was detected for the IL6 promoter variant rs1800795 or the IL1B promoter variant rs16944 [14]. Immune-cell recognition pathways have also been examined. In a Chinese Han case–control study of 106 patients and 220 controls, KIR2DS2 was more frequent in severe DED, and the combination of KIR2DS2 with the HLA-C1 allele group was associated with severe disease (OR 2.083), while HLA-C allele groups considered independently did not differ significantly between cases and controls [15].

These small, ancestry-specific candidate-gene studies generate hypotheses regarding cytokine and innate immune pathways but do not establish these pathways as inherited determinants of DED and require independent replication.

Genetic variation may also influence the ocular-surface response to a defined environmental or iatrogenic challenge. In 143 active-duty U.S. Army personnel undergoing refractive surgery, three THBS1 variants were associated with subsequent chronic keratoconjunctivitis. The strongest association involved rs1478604, for which being a carrier of the minor allele was associated with approximately 2.5-fold greater odds of chronic ocular-surface inflammation. Carriers also showed lower ocular-surface expression of thrombospondin-1 and higher IL-1β expression after surgery [16]. The study therefore provides an example in which genotype was associated not simply with cross-sectional DED status but with persistence of inflammation following a specific ocular insult. Its relevance is nevertheless primarily to post-refractive-surgery inflammation and should not be extrapolated directly to idiopathic DED.

A Chinese Han candidate-gene study including 124 DED cases and 135 controls reported associations of VDR Apa-1 and Taq-1 polymorphisms with DED [17]. Given the modest ancestry-specific sample, candidate-gene design, and lack of independent replication, these findings should be regarded as exploratory.

Other inflammatory candidate-gene studies have produced negative or equivocal results. In an Israeli cohort comparing 82 patients with Sjögren syndrome and 98 with non-Sjögren dry eye, adjusted analyses did not identify significant associations for TNF, IL10, or TNFAIP3 variants [18]. Likewise, in 328 predominantly male U.S. veterans, TNFR1 rs1800693 did not materially distinguish DED symptoms or most ocular-surface signs, and a treatment-response difference observed in a small subgroup was not statistically significant [19]. Collectively, these findings emphasize that biological plausibility is insufficient without adequately powered independent replication.

More recent HLA studies have provided additional evidence that immune–genetic associations may differ according to DED endotype. In a cross-sectional study of moderate-to-severe DED, Chen et al. evaluated 64 patients with non-Sjögren DED and 40 with Sjögren DED against population HLA reference data. HLA-B40:01 was more frequent in the non-Sjögren group than in the reference population (OR 2.42), and being a carrier was associated with greater ocular-surface staining and a higher proportion of severe disease. In Sjögren DED, increased frequencies were identified for HLA-B40:01, HLA-C07:02, HLA-DQB106:01, and HLA-DRB1*16:02 [20].

These findings should be considered preliminary because the clinical subgroups were small and were compared with population HLA reference frequencies rather than a concurrently recruited control group. Independent replication is therefore required before these alleles can be regarded as established DED susceptibility factors.

The distinction between general DED and Sjögren disease becomes particularly important when the strength of the underlying genetic evidence is considered. Sjögren disease is a systemic autoimmune disorder in which lymphocytic involvement of the lacrimal and salivary glands produces clinically important keratoconjunctivitis sicca and xerostomia [21].

Its immunogenetic basis has been investigated in substantially larger cohorts than nonspecific DED, although it remains incompletely characterized and polygenic, with genetic variation conferring susceptibility rather than direct causation.

A large association study identified an exceptionally strong signal across the HLA region together with genome-wide significant associations at IRF5–TNPO3, STAT4, IL12A, FAM167A–BLK, DDX6–CXCR5, and TNIP1, linking susceptibility to both innate and adaptive immune pathways [22]. A subsequent GWAS in individuals of European ancestry expanded this architecture by identifying ten additional genome-wide significant regions, including loci containing or neighboring CD247, PTTG1–MIR146A, PRDM1–ATG5, TNFAIP3, TYK2, and RPTOR, among others. Integration with expression and regulatory datasets further connected several associated variants to gene regulation in immune cells and salivary gland tissue [23].

Genetic heterogeneity is present even within Sjögren disease itself. In a multicenter analysis involving 918 patients and 1264 controls, with replication in an additional 177 patients and 7672 controls, Thorlacius et al. separated Sjögren disease into clinically and genetically distinct groups according to SSA/SSB antibody status. A strong HLA-DQA1 association was observed in the SSA/SSB-positive subgroup (OR 6.10), whereas the corresponding association was absent in antibody-negative disease; additional independent MHC class I and II signals were likewise concentrated in seropositive patients [24]. Contemporary genetic and epigenetic analyses consequently describe Sjögren disease as a complex immune-mediated phenotype in which HLA and non-HLA susceptibility, interferon-related pathways, lymphocyte regulation, epigenetic state, sex, and environmental context interact rather than operating as isolated genetic effects [25,26,27].

Genetic studies of Sjögren disease provide stronger evidence for inherited immune susceptibility in a systemic autoimmune disorder that includes ocular dryness, but they should not be used as a proxy for the genetic architecture of DED as a whole. Candidate-gene studies in DED raise hypotheses involving mucin biology, cytokine signaling, immune-cell recognition, and inflammatory regulation, but most associations remain small, ancestry-specific, or unreplicated [12,13,14,15,16,17,18,19,20]. HLA and non-HLA associations in Sjögren disease are supported by larger genomic studies [21,22,23,24,25,26,27], although Sjögren itself remains a complex polygenic disorder. This contrast primarily illustrates the need for larger, phenotype-specific, and independently replicated genomic studies in DED.

### 3.3. Genome-Wide Associations, Polygenic Risk, Pleiotropy, and Gene–Environment Architecture

Genome-wide studies have begun to identify loci associated with specific DED phenotypes and ascertainment definitions, although the strength and maturity of the evidence differ substantially among studies. In the Taiwan Biobank, a GWAS of Han Chinese participants reported multiple DED-associated loci, including DNAJB6, MAML3, DCHS1, MUC16, ZFPM2, GAS2L3, LINC02267, SIRPB3P, and HULC [28]. MUC16 is of particular biological interest because it encodes a membrane-associated mucin contributing to ocular-surface glycocalyx barrier function. A substantially larger multi-ancestry analysis from the Million Veteran Program reported ten genome-wide significant loci across nine susceptibility regions, including BLK, GATAD2A, MLLT10, FAM180B, EPHA5, GRIA1, and SYNGAP1; however, at the time of the present literature search this study was available only as a medRxiv preprint and had not undergone peer review, and its findings should therefore be considered preliminary pending peer-reviewed publication and independent replication [29]. A separate GWAS focused specifically on neuropathic ocular pain rather than DED as a general clinical syndrome. In 329 participants, rs140293404 reached genome-wide significance (*p* = 1.23 × 10^−8^), whereas ten additional SNPs reached suggestive significance; this finding has not yet undergone independent replication [30].

Based on data provided by the associative SNPs identified in GWAS, polygenic risk stratification (PRS) tools for DED can be constructed. These are calculated as a weighted sum of the individual’s genetic variants, using a weight corresponding to the estimated effect of each individual allele. Newer PRSs can include hundreds to millions of variants, even those with smaller individual effects, and it is intended to summarize the combined effect of these.

In the Taiwan Biobank study, a higher PRS was associated with greater DED risk [28]. However, association with disease status did not translate into strong discrimination when the genetic score was used in isolation. The AUROC of the PRS alone was 0.537 in participants younger than 60 years and 0.509 in those aged 60 years or older; incorporation of age, sex, body mass index, depression, and manic episodes increased the AUROC to 0.648 and 0.600, respectively [28]. These models were assessed using internal five-fold cross-validation rather than an independent external cohort. Thus, the study provides evidence that polygenic information is associated with DED risk, but it does not yet establish sufficiently accurate, calibrated, or externally validated prediction for clinical use.

Another interesting aspect is pleiotropy, evaluating how much the genetic component of DED is shared with other phenotypes. In particular, conditions such as fibromyalgia (rG = 0.69), chronic multisite pain (rG = 0.64), post-traumatic stress disorder (rG = 0.58), ADHD (rG = 0.45), major depressive disorder (rG = 0.36), and anxiety (rG = 0.33) showed a genetic component shared with DED. Furthermore, some genetic components of DED appear to be linked to an immunological predisposition for Sjögren’s syndrome, systemic sclerosis, systemic lupus erythematosus, and rheumatoid arthritis [29]. A positive genetic correlation with DED has been demonstrated in depression through a study conducted using Taiwan Biobank and UK Biobank. Among the common functional genes, MUC16 and TENM2 emerged, in which in both cases they were different variants located in the same gene and not the same SNP that directly determined both conditions [31].

The same study used Mendelian randomization (MR) in an attempt to establish whether the association between DED and depression could be due to a causal relationship, allowing us to identify a bidirectional relationship: genetic predisposition to DED was associated with the risk of depression (Tawainan Biobank OR = 1.877, 95% CI: 1.447–2.435; UK Biobank OR = 1.113, 95% CI: 1.055–1.175) and conversely genetic predisposition to depression was associated with the risk of DED (Taiwainan Biobank OR = 1.104, 95% CI: 1.049–1.162; UK Biobank OR = 1.659, 95% CI: 1.301–2.116). Bidirectionality persisted even after using COJO to reduce the effect of disequilibrium linkage [31].

Mendelian randomization has also been useful for studying the relationship with modifiable behavioral and environmental factors, such as cigarette smoking. Genetic predisposition to lifetime smoking (OR = 1.757; 95% CI 1.021–3.025), and to regular smoking OR = 2.121; 95% CI 1.017–4.423) was associated with an increased risk of DED, while no statistically significant associations emerged for smoking intensity and smoking cessation. These findings support a possible causal relationship between genetic predisposition to cigarette smoking and DED [32].

Clinical utility of a PRS requires more than an association with disease status. A clinically useful model should demonstrate adequate discrimination, calibration of predicted versus observed risk, incremental predictive value beyond conventional clinical factors, and independent external validation. None of these requirements can be assumed from performance within the discovery cohort alone. In addition, PRS accuracy strongly depends on the ancestral representation of the populations used in the GWAS from which the score is derived.

Most GWASs were developed on populations of European ancestry, resulting in higher PRS accuracy on samples genetically closer to these, while a reduction in accuracy is observed as one moves away from the GWAS of origin [33]. Transferability, that is the ability of a PRS to maintain its accuracy when applied outside the population in which it was developed, does not vary only among large ancestral categories. Analysis of 84 complex traits showed that the accuracy of PRSs progressively decreases as the genetic distance from the population used for training increases, even among individuals of European ancestry but more genetically distant from the sample used, thus also underlining the importance of individual genetic variability [34]. The performance obtained in the original cohort cannot be considered sufficient to establish the generalizability and reproducibility of a PRS. In this regard, the Polygenic Score Catalog (PGS Catalog) was developed, an open-access resource that collects and organizes PRSs available in the literature, providing the data and information needed to interpret them, reproduce them, apply them to other datasets, and evaluate their performance [35].

After considering inherited susceptibility and polygenic models, it is also important to examine potential gene–environment interactions.

A Taiwan Bank study of 129,451 individuals of Chinese Han ancestry, including 4517 residing in geographic areas near a hot spring, analyzed the interaction between polygenic predisposition and residence in such areas. If in a first analysis not adjusted for confounding factors such as demographic, environmental and socio-economic factors, the prevalence of DED was higher in thermal areas, in subsequent adjusted analyses the prevalence was lower (OR = 0.81).

Gene–environment interaction analysis showed that in subjects without high genetic risk, hot-spring residence was associated with a lower risk of DED, while the simultaneous presence of genetic predisposition and spa residence was associated with a higher risk. PRSs constructed on the population residing in thermal areas (AUC 0.9168) and that in non-thermal areas (AUC 0.5015) demonstrate that the same genetic variants do not have the same predictivity in different environments. The marked difference in predictive performance between the hot-spring and non-hot-spring subgroups was interpreted as possible environment-dependent modification of PRS performance [36]. However, although the analyses adjusted for several demographic, environmental, and socioeconomic variables, residual confounding, exposure misclassification, and incomplete adjustment for other DED risk factors cannot be excluded. The finding should therefore be regarded as hypothesis-generating rather than as evidence of a causal gene–environment interaction.

The DryEyeRhythm prospective protocol proposes integrating Tohoku Medical Megabank genetic information with longitudinal digital phenotyping and environmental, lifestyle, and demographic variables. Because this is a protocol study, it currently provides no evidence for gene–environment associations in DED [37].

As previously stated, the studies available to date still highlight several limitations in the genetic characterization of DED. A first limitation is represented by the marked phenotypic heterogeneity of DED, which includes signs and clinical symptoms that are not uniformly collected. This aspect could influence the identification of genetic associations and make the replication of results between independent cohorts more complex. For this reason, it would be appropriate to develop more homogeneous clinical criteria for future genomic studies [29,36] and in this context digital phenotyping can be a valid tool [37]. Another limitation already illustrated concerns the differences in ancestry of the populations examined and, consequently, the transferability of genetic results [33]. There also remains a need for external replication and validation. A high PRS in the study cohort alone is not enough to demonstrate its generalizability, so the Polygenic Score Catalog was developed precisely to try to improve this aspect [35].

The principal genomic findings, pleiotropic associations, gene–environment interactions, and current limitations of polygenic risk scores are summarized in Table 1.

The principal primary genetic studies discussed in this review are compared in Table 2, with particular attention to phenotype definition, study design, evidence strength, and replication status.

### 3.4. Functional Genomics, Epigenetics, and Single-Cell Resolution Toward Precision Ocular Surface Medicine

Functional interpretation represents a major challenge in transforming a genetic association into a biological mechanism and, potentially, into a therapeutic target. Importantly, most transcriptomic, epigenetic, non-coding RNA, and single-cell studies discussed below characterize disease-associated molecular states rather than inherited susceptibility. Unless a molecular alteration is directly linked to germline variation, it should not be interpreted as a genetic risk factor for DED.

A DED statistically associated variant does not necessarily identify the causal gene, and when a gene of interest is pinpointed, this does not establish in which tissue, cell type, or disease state its effect manifests. For this reason, data from GWAS and SNP studies must be integrated with functional genomics approaches to understand how genetic susceptibility translates into changes in gene expression and biological pathways. Single-cell atlases of the human cornea and adjacent conjunctiva, as well as the lacrimal gland, have provided reference maps of their cellular composition, distinguishing epithelial, stromal, endothelial, immune, and limbal progenitor populations in the cornea and identifying the major acinar and ductal compartments of the lacrimal gland [38,39]. Ocular surface transcriptomics has already shown that DED is associated with complex molecular signatures and not a single alteration, with transcriptional and immune programs that may be shared between some phenotypes or differ in other clinical contexts [40,41,42]. Inflammatory networks strongly associated with HLA-DRA/HLA-DRB1 expression have been identified in the conjunctiva of patients with the disease, involving interferon signaling, TNF, Toll-like receptors, complement, chemokines/cytokines, STATs, and MAPK pathways [40]. Other transcriptional profiles have highlighted how two conditions that may appear clinically similar, such as DED and ocular surface disorder associated with topical therapy for glaucoma, can exhibit different molecular profiles [41].

Bulk transcriptomic analyses can identify tissue-level expression patterns but generally cannot assign those patterns to specific cell populations.

In this context, single-cell RNA sequencing (scRNA-seq) and single-nucleus RNA sequencing (snRNA-seq) approaches have made it possible to localize these signals to specific cell types and states, constructing reference maps of specific tissues. The application of these methodologies to DED models has allowed researchers to attribute specific pathological pathways to defined cell populations: in the cornea subjected to desiccating stress, scRNA-seq has indeed revealed a significant remodeling of the corneal immune component with an increase in resident macrophages from 18.2% to 55.2%, accompanied by a transcriptional reconfiguration and an increase in inflammatory and neurosensitizing mediators, including CXCL1, thereby associating macrophage activation with the sensorineural alterations of DED [43]. In the conjunctiva of murine models of dry eye desiccating stress, the integration of scRNA-seq and single-cell assay for transposase-accessible chromatin sequencing (scATAC-seq) further enabled the distinction of different populations of mononuclear phagocytes: TLF-CCR2+, predominantly associated with inflammatory programs, and TLF+CCR2-, predominantly associated with phagocytic and immunoregulatory programs and including MRC1+ macrophages. Experimental depletion of MRC1+ macrophages led to increased inflammation and worsening ocular surface damage, providing further functional support for the regulatory role of this cell population [44]. In the lacrimal gland of an experimentally induced mouse model of DED, snRNA-seq allowed for the localization alterations to specific cellular compartments, highlighting a reduction in acinar cells and their secretory potential, an increase in ductal and myoepithelial cells, and intensification of the immune response, associated with an altered interaction between immune cells and parenchymal cells [45].

These experimental studies illustrate how single-cell approaches can localize disease-associated signals to defined cell populations, but their relevance to human DED susceptibility remains indirect because most available data derive from experimental models and do not establish germline genetic effects.

Epigenetic studies provide a further level of molecular characterization by examining regulatory changes in gene expression that may arise in association with disease state, environmental exposure, or other biological influences. Such changes should not be considered inherited susceptibility factors unless they are directly connected to germline genetic variation.

In mouse models of experimental DED, an association has been observed between time-dependent upregulation of the circadian PER2 and PER3 genes and hypomethylation of their respective promoters. Furthermore, silencing of PER2 and PER3 reduces the expression of inflammatory factors in vitro, supporting a possible role for DNA methylation in the epigenetic regulation of circadian genes and the inflammatory response in experimental DED [46].

Similarly, the integration of reduced representation bisulfite sequencing (RRBS) and RNA-seq in the lacrimal glands of mouse models of Sjögren’s syndrome (SS)-associated dry eye revealed a remodeling of DNA methylation, characterized by a global increase in methylation, but a concomitant locus-specific hypomethylation of promoters of upregulated genes, including Irf4 and Itgal, involved in the T-cell immune response. These results must, however, be interpreted with caution, given the murine nature of the model, the need for further mechanistic confirmation, and the analysis of the entire lacrimal gland without distinguishing among individual cell types [47].

In an observational study conducted on DED patients and healthy controls, 32 differentially expressed miRNAs were identified, with increased expression of miR-450b-5p, miR-1283 and miR-3671 in the tears of DED patients; these miRNAs were subsequently validated in vitro as negative regulators of TGF-β2, and the combination of the three miRNAs with TGF-β2 also showed a high discriminatory capacity between patients and controls [48]. These tear miRNA alterations represent disease-associated expression profiles and potential molecular biomarkers rather than evidence of inherited genetic susceptibility. More broadly, non-coding RNAs (ncRNAs), including miRNAs, lncRNAs and circRNAs, have been implicated in the modulation of inflammation, immune responses, regulated cell death, and epithelial barrier integrity in DED; however, evidence for lncRNAs and circRNAs remains emerging and requires further validation [49,50].

Overall, transcriptomic, epigenomic, and ncRNA studies provide complementary information on disease-associated molecular regulation and tissue states. Their principal value in the present context is to help interpret biological pathways and cellular mechanisms that may intersect with inherited susceptibility; the molecular alterations identified by these approaches should not themselves be classified as germline genetic risk factors unless a direct genotype–molecular phenotype relationship is demonstrated.

The advanced example of the transition from mechanism to therapeutic intervention is represented by investigation on miR-328. Benzalkonium chloride (BAC) and hyperosmotic stress induced an upregulation of miR-328 in corneal cells, while in experimental animal models of BAC-induced DED, topical treatment with anti-miR-328 reduced corneal staining and apoptosis and promoted epithelial recovery, supporting the potential of miR-328 as a preclinical therapeutic target [51]. This strategy subsequently progressed to clinical trials with SHJ002, an anti-miR-328 antisense oligonucleotide, which in a multicenter, randomized, double-blind, vehicle-controlled phase 2 clinical trial conducted in 85 patients with DED, resulted in a significantly greater reduction in total corneal fluorescein staining (tCFS) compared to the vehicle, showing a favorable tolerability profile [52].

This result represents an example of the clinical translation of a molecular target, but not yet of pharmacogenomics or genotype-guided precision medicine, from which we are still far.

At present, no evidence supports genotype-guided treatment selection in DED. Molecular biomarkers and therapeutic targets may inform mechanistic research, but their use for individualized treatment selection remains investigational.

Single-cell approaches also retain methodological limitations, including loss of spatial information and the need to validate some identified cell states or clusters, which currently restrict direct clinical extrapolation [53].

Evidence from the molecular characterization of DED is guiding its progressive clinical translation, especially through the identification of biomarkers and potential therapeutic targets, although the maturity level of the different applications remains heterogeneous. An example of a disease-associated ocular surface molecular biomarker, distinct from the germline HLA allele associations discussed above, is conjunctival HLA-DR expression: in 205 patients with moderate-to-severe DED enrolled in the NORTHERN LIGHTS trial, its conjunctival expression showed wide interindividual variability and was significantly associated with corneal fluorescein staining, but not with the other signs and symptoms assessed, suggesting that a single biomarker may reflect specific aspects of ocular surface damage rather than the overall severity of DED [54].

**Figure 1:**
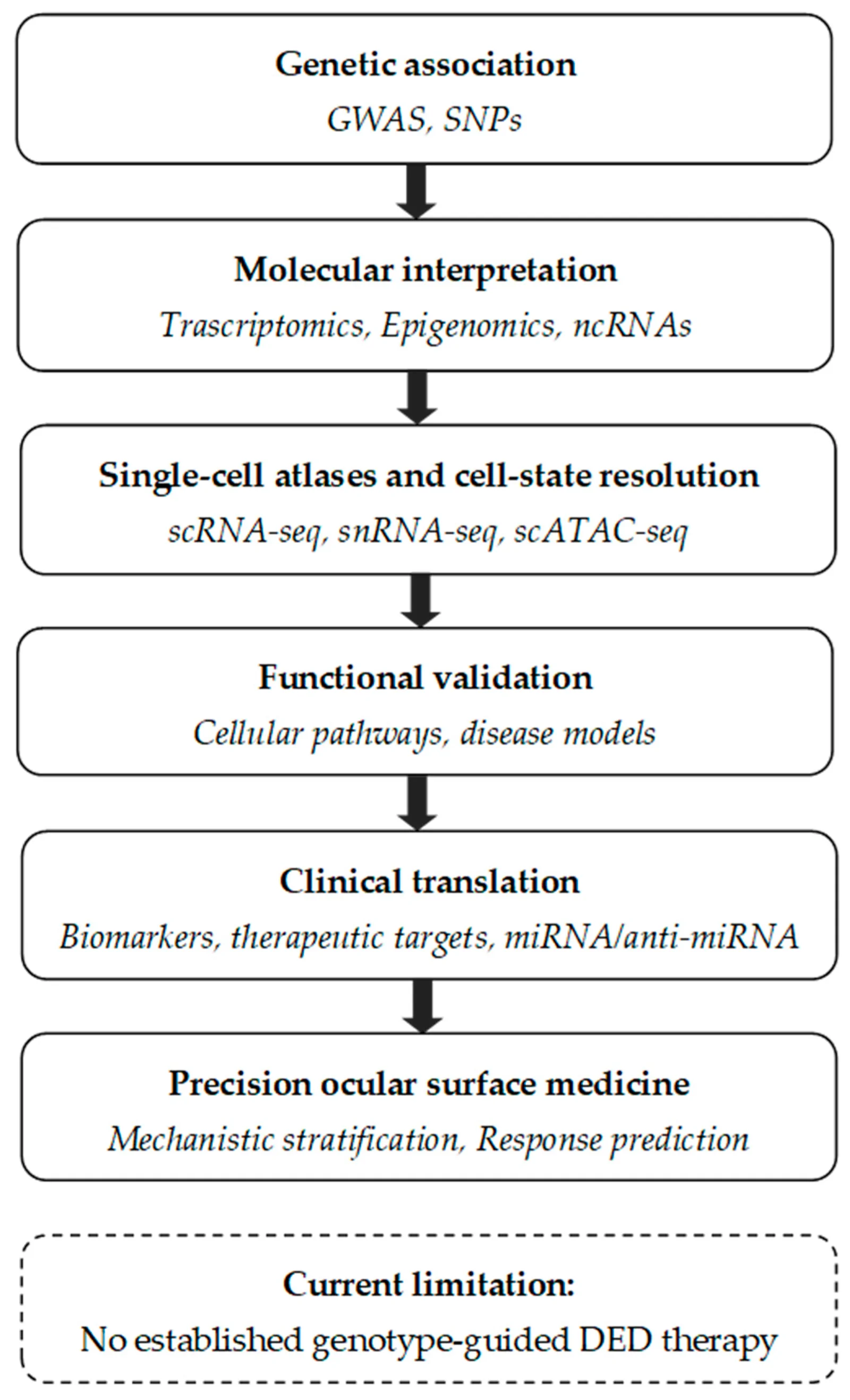
From genetic associations to precision medicine of the ocular surface in D. GWAS, genome-wide association study; SNP, single-nucleotide polymorphism; DED, dry eye.

Figure 1 outlines the proposed pathway from genetic associations to precision medicine in DED.

## 4. Discussion

### 4.1. Strength of Genetic Evidence and the Problem of DED Phenotype Definition

Current evidence suggests that genetic susceptibility may contribute to DED together with environmental, systemic, ocular-surface, and immune factors, but the magnitude and clinical relevance of this contribution remain uncertain. Twin studies and emerging biobank-scale analyses provide the most direct evidence, whereas most candidate-gene studies are small, ancestry-specific, and unreplicated. These candidate associations should therefore be considered hypothesis-generating rather than established DED susceptibility loci. The priority for the field is not to identify isolated genes associated with broad DED labels, but to identify reproducible genetic susceptibility signals within standardized and biologically meaningful phenotypes.

A central problem is the phenotype itself. The historical evolution from TFOS DEWS II to TFOS DEWS III reflects increasing recognition that DED cannot be defined by a single sign or mechanism [1,2,3,55,56,57]. Tear film instability, hyperosmolarity, inflammation, epithelial damage, gland dysfunction, and neurosensory abnormalities may coexist in different combinations. Epidemiological studies have likewise shown marked variability according to the definition applied [4,57]. A GWAS based on self-reported physician diagnosis, for example, is not genetically equivalent to a study of severe aqueous-deficient DED defined by Schirmer testing and staining, nor to a cohort selected for meibomian gland dysfunction or neuropathic-like ocular pain.

Phenotypic heterogeneity is a plausible contributor to the modest and inconsistent genetic signals reported to date. A major priority is therefore the use of standardized and reproducible diagnostic criteria together with explicit classification of clinically relevant DED phenotypes and endotypes. Future genomic studies should consistently report standardized symptom measures together with tear-film stability, tear production, meibomian-gland parameters, ocular-surface staining as a measure of epithelial damage or disease severity, and, where relevant, sensory measures. Such standardization would reduce phenotype misclassification and allow investigators to test whether specific dimensions of DED have distinct genetic associations.

### 4.2. Sensory and Immunogenetic Heterogeneity

The symptom–sign discrepancy deserves particular attention. Twin studies linking DED with pain sensitivity and shared genetic determinants of chronic pain syndromes [10,11], together with emerging genomic findings in neuropathic ocular pain [30] and provisional large-scale DED data implicating neuronal pathways [29], suggest that sensory biology may contribute to genetic heterogeneity.

The available data therefore support only the possibility that sensory traits contribute to heterogeneity in symptom burden; they do not establish a primary neurological mechanism for DED. Future studies should evaluate sensory phenotypes alongside tear-film and ocular-surface measures rather than treating these mechanisms as competing explanations.

A future precision framework may therefore need to distinguish inflammatory, glandular, evaporative, and neurosensory dimensions within individual patients.

The comparison between common DED and Sjögren disease further demonstrates the importance of endotyping. Sjögren disease has a reproducible immune–genetic architecture involving the HLA region, interferon-associated pathways, B-cell regulatory genes, and other immune loci [22,23,24,25,26,27]. These data provide strong mechanistic evidence for genetically determined immune susceptibility in autoimmune lacrimal gland disease. However, using Sjögren loci as surrogate DED loci would introduce substantial biological misclassification. The more appropriate strategy is to determine which immune–genetic pathways are shared across DED endotypes and which remain specific to systemic autoimmunity.

### 4.3. Genome-Wide Evidence, Polygenic Risk, and Gene–Environment Interactions

Direct GWAS evidence for general DED remains insufficient for immediate clinical translation. The Taiwan Biobank study demonstrates the feasibility of deriving a DED PRS but also illustrates the gap between statistical association and clinical prediction: discrimination of the PRS alone was close to chance and improved only modestly after incorporation of conventional covariates [28]. Calibration, clinically meaningful incremental predictive value over established clinical risk factors, decision-analytic utility, and independent external validation remain insufficiently established for DED PRSs.

The much larger Million Veteran Program analysis is potentially transformative because of its sample size, multi-ancestry design, and identification of both neural and immune pathways, but its preprint status requires that its conclusions remain provisional until peer-reviewed publication and external replication [29]. Consequently, current PRS should be considered research tools rather than clinical diagnostic tests.

Ancestry diversity is another major limitation. Many early candidate studies were conducted in individual Asian or European-derived populations, while broader genomic medicine has demonstrated substantial deterioration in PRS performance when discovery and target populations differ genetically [33,34]. This limitation is not simply methodological; deploying poorly transferable scores could amplify existing disparities in access to precision medicine. DED genomics therefore requires deliberate recruitment of ancestrally diverse populations, transparent reporting of ancestry and population structure, and independent external validation.

Gene–environment interaction is likely to be especially relevant to ocular surface disease. Unlike many internal organs, the ocular surface is directly and continuously exposed to humidity, airflow, pollutants, digital-device behavior, contact lenses, topical medications, cosmetics, surgery, sleep-related exposure, and other modifiable factors [5]. A genetic variant may therefore exert little measurable effect under baseline conditions but become clinically relevant after chronic environmental stress or injury.

The THBS1 study following refractive surgery provides only preliminary and context-specific support for this concept because of its modest sample size and the difficulty of separating genetic effects from surgical, environmental, and host-related contributors to postoperative ocular-surface disease [16].

Integrating genomic data with longitudinal exposome measurements and digital phenotyping may uncover interactions that conventional cross-sectional GWAS cannot detect [37].

### 4.4. Functional Molecular Evidence and Limits of Clinical Translation

Functional interpretation represents the next major challenge. A statistically associated variant does not identify a causal gene, and a causal gene does not reveal the cell type or disease state in which its effect is expressed. Human corneal and lacrimal single-cell atlases [38,39], conjunctival transcriptomics [40,41,42], and experimental single-cell datasets [43,44,45] provide increasingly detailed cellular maps through which future DED loci can be interpreted.

Future studies should first establish replicated DED-associated loci and then determine whether these loci map to gene-expression or regulatory changes in relevant ocular-surface cell types before undertaking functional validation.

Epigenetic and non-coding RNA studies provide an additional bridge between inherited susceptibility and the environment. However, causality remains difficult to establish because inflammation itself can alter DNA methylation and RNA expression. Cross-sectional molecular differences between DED and control tissues should therefore not be described as genetic risk factors unless an inherited variant is directly demonstrated. Longitudinal studies and genetically informed approaches will be required to distinguish causal regulatory mechanisms from biomarkers of established disease.

The emerging therapeutic literature also requires careful terminology. The phase 2 anti-miR-328 trial represents a notable step toward molecularly targeted ocular surface therapy [52], but it is not yet an example of pharmacogenomics or genotype-guided precision medicine. True genomic precision therapy would require demonstration that molecular or inherited patient characteristics predict treatment selection or response. In the nearer term, genetics may be more useful for mechanistic stratification, biomarker discovery, or identification of new therapeutic targets than for individual clinical risk prediction.

## 5. Conclusions

As per the current evidence, genetic susceptibility may play a role in the development of dry eye disease (DED) as one component of a multifactorial disorder. However, the extent of this contribution and its clinical significance remain uncertain. Evidence from twin studies and emerging genome-wide analyses is the most direct, while many candidate-gene associations remain small, ancestry-specific, and unreplicated. The strongest immunogenetic framework for autoimmune ocular dryness has been identified in Sjögren disease; however, one should not generalize its complex polygenic architecture to DED overall. Present polygenic risk scores display limited discrimination and lack sufficient external validation for clinical application. Moreover, disease-associated changes in transcriptomics, epigenetics, and non-coding RNAs should not be construed as inherited risk factors without direct evidence in the germline.

These limitations present several priorities. The definitions and assessments of DED should be standardized, clinically relevant phenotypes and endotypes should be explicitly reported, and future genomic studies should utilize substantially larger and much more ancestrally diverse cohorts with independent replication. Candidate-gene findings should remain hypothesis-generating unless confirmed in adequately powered datasets. The interpretation of genomic results should include environmental exposures together with tissue- or cell-specific functional data. This integration, however, should currently be regarded as mechanistic research and not established precision medicine. Polygenic risk scores or molecular classifiers should be used in routine patient care only after robust replication, independent external validation, and proof of clinically relevant incremental value over conventional clinical factors.

## Figures and Tables

**Table 1:** Summary of the principal genome-wide genetic findings, pleiotropic associations, gene–environment interactions, and current limitations of polygenic risk scores in dry eye disease.

Domain	Main Findings
Main genes and loci identified	Peer-reviewed GWAS of biobank-defined DED reported loci including DNAJB6, MAML3, LINC02267, DCHS1, SIRPB3P, HULC, MUC16, GAS2L3, and ZFPM2 [28]. A large multi-ancestry Million Veteran Program preprint reported additional loci including BLK, MLLT10, FAM180B, GATAD2A, EPHA5, GRIA1, and SYNGAP1; these findings remain preliminary pending peer-reviewed publication and external replication [29]. In a separate GWAS of neuropathic ocular pain, rs140293404 reached genome-wide significance but has not yet been independently replicated [30].
Genetic correlation and pleiotropy	Positive genetic correlations were reported with fibromyalgia (*r*g = 0.69), chronic multisite pain (*r*g = 0.64), post-traumatic stress disorder (*r*g = 0.58), attention-deficit/hyperactivity disorder (*r*g = 0.45), major depressive disorder (*r*g = 0.36), and anxiety (*r*g = 0.33), supporting pleiotropic relationships between DED and pain-related and neuropsychiatric phenotypes [29].
Gene–environment interaction	In the Taiwan Biobank, a crossover gene–environment interaction was observed between genetic predisposition to DED and residence in areas with hot springs [36].
Limits of PRSs	Current PRSs are limited by ancestry bias, reduced transferability across genetically different populations, and the need for independent external validation [33,34,35].

Abbreviations: DED, dry eye disease; GWAS, genome-wide association study; PRS, polygenic risk score; *r*g, genetic correlation coefficient.

**Table 2:** Principal primary genetic association and genomic studies relevant to dry eye disease and selected dry eye endotypes.

Study	Population/Ancestry	Sample Size	Phenotype/Endotype	Design	Principal Finding	Effect Estimate/Statistical Evidence	Evidence/Replication Status	Major Limitations
Imbert et al. [13]	Female Caucasian participants	Small clinical cohort	Non-Sjögren aqueous-deficient DED and evaporative DED	Candidate-gene association	MUC1 rs4072037 genotype frequencies differed between controls and both DED subtypes; ERα association was not reproduced	MUC1: *p* = 0.017 for aqueous-deficient DED; *p* = 0.015 for evaporative DED	Candidate-gene finding; not independently established at genome-wide level	Small ancestry-specific cohort; candidate-gene design; no large independent replication
Na et al. [14]	Korean	251 DED; 109 controls	Non-Sjögren DED	Candidate-gene case–control study	Associations reported for IL1B rs1143634 and IL6R rs8192284	IL1B C/T: OR 3.337; IL6R C/C: OR 2.12	Candidate-gene findings; unreplicated	Single-ancestry cohort; multiple candidate variants; no independent replication
Ren et al. [15]	Chinese Han	106 DED; 220 controls	DED; severe DED subgroup	Candidate KIR/HLA case–control study	KIR2DS2 and KIR2DS2/HLA-C1 combination associated with severe DED	KIR2DS2: OR 1.828; KIR2DS2/HLA-C1: OR 2.083	Candidate-gene finding; unreplicated	Small cohort; subgroup analysis; ancestry-specific
Contreras-Ruiz et al. [16]	U.S. active-duty Army personnel	143	Post-refractive-surgery chronic keratoconjunctivitis	Retrospective genetic cohort	THBS1 variants associated with chronic postoperative ocular-surface inflammation	rs1478604: OR 2.5, 95% CI 1.41–4.47; rs2228262/rs2292305: OR 1.9	Context-specific candidate-gene association; unreplicated	Iatrogenic phenotype; small selected population; not generalizable to idiopathic DED
Meng et al. [17]	Chinese Han	124 DED; 135 controls	DED	Candidate-gene case–control study	VDR Apa-1 and Taq-1 polymorphisms associated with DED	Apa-1 aa: OR 2.803, 95% CI 1.350–5.820; Taq-1 tt: OR 0.362, 95% CI 0.141–0.930	Candidate-gene finding; unreplicated	Single ancestry; modest sample size; potential environmental confounding
Ben-Eli et al. [18]	Israeli cohort	82 Sjögren; 98 non-Sjögren dry eye	Sjögren vs. non-Sjögren dry eye	Candidate-gene comparison	No robust adjusted association for TNF, IL10, or TNFAIP3 variants	Nominal TNF difference in additive analysis; associations lost after adjustment	Negative/equivocal candidate-gene evidence	No concurrently recruited healthy control group; modest sample size
Acuna et al. [19]	U.S. veterans; predominantly male	328	DED symptoms/signs	Prospective candidate-gene cohort	TNFR1 rs1800693 did not materially distinguish DED phenotype; treatment-response trend was nonsignificant	Treatment subgroup: *p* = 0.22	Negative candidate-gene evidence	Predominantly male veteran cohort; small treated subgroup
Chen et al. [20]	Clinical DED cohort; population HLA reference comparator	64 non-Sjögren DED; 40 Sjögren DED	Moderate-to-severe non-Sjögren and Sjögren DED	Cross-sectional HLA association study	HLA-B*40:01 enriched in non-Sjögren DED; additional HLA associations in Sjögren DED	Non-Sjögren HLA-B x 40:01: OR 2.42, *p* < 0.001	Recent peer-reviewed association; independent replication required	Small clinical cohorts; population reference rather than concurrently recruited controls
Hsu et al. [28]	Taiwan Biobank; Han Chinese	40,112 total; 14,185 DED and 25,927 controls	Biobank-defined DED	GWAS and PRS study	Multiple DED-associated loci; PRS associated with DED risk	PRS-alone AUROC 0.537 (<60 y) and 0.509 (≥60 y); PRS plus covariates 0.648 and 0.600	Peer-reviewed GWAS; internal cross-validation; no independent external PRS validation	Single ancestry; phenotype ascertainment; limited PRS discrimination; external validation lacking
Gorman et al. [29]	Million Veteran Program; multi-ancestry	132,657 cases; 352,201 controls	Algorithm-defined DED	Multi-ancestry GWAS and genetic-correlation analysis	Ten genome-wide significant loci in nine regions; neural and immune pathways implicated	Genome-wide significant loci reported; genetic correlations with systemic phenotypes	Preprint; not peer reviewed at literature-search cutoff	Preliminary evidence; veteran population; phenotype based on diagnosis/prescription algorithm; external replication required
Huang et al. [30]	U.S. veterans	329	Neuropathic ocular pain	GWAS	rs140293404 reached genome-wide significance	*p* = 1.23 × 10^−8^	Genome-wide significant discovery finding; not independently replicated	Small cohort; neuropathic ocular pain rather than general DED; ancestry/generalizability limitations
Chang et al. [31]	Taiwan Biobank and UK Biobank	Large East Asian and European biobank datasets	DED and depression	Cross-biobank GWAS/genetic correlation and bidirectional MR	Shared genetic architecture and bidirectional MR associations reported between DED and depression	DED → depression and depression → DED associations reproduced across TWB and UKB analyses	Peer-reviewed cross-biobank genetic evidence; MR inference rather than direct clinical causality	Phenotype definitions differ between biobanks; MR assumptions apply; findings do not establish individual-level causation
Yang et al. [32]	European-ancestry GWAS datasets	Summary-level GWAS datasets	DED and smoking-related exposures	Two-sample Mendelian randomization	Genetic liability to lifetime and regular smoking associated with increased DED risk	Lifetime smoking: OR 1.757; regular smoking: OR 2.121	Peer-reviewed MR evidence	Indirect genetic-instrument evidence; ancestry restriction; MR assumptions and instrument validity

Abbreviations: DED, dry eye disease; GWAS, genome-wide association study; HLA, human leukocyte antigen; KIR, killer cell immunoglobulin-like receptor; MR, Mendelian randomization; OR, odds ratio; PRS, polygenic risk score.

## Data Availability

No new data were created or analyzed in this study. Data sharing is not applicable to this article.
